# Cationic Phenosafranin Photosensitizers Based on Polyhedral Oligomeric Silsesquioxanes for Inactivation of Gram-Positive and Gram-Negative Bacteria

**DOI:** 10.3390/ijms222413373

**Published:** 2021-12-13

**Authors:** Krystyna Rozga-Wijas, Irena Bak-Sypien, Katarzyna Turecka, Magdalena Narajczyk, Krzysztof Waleron

**Affiliations:** 1 Centre of Molecular and Macromolecular Studies, Polish Academy of Science, Sienkiewicza 112, 90-363 Lodz, Poland; sypieni@cbmm.lodz.pl; 2 Department of Pharmaceutical Microbiology, Faculty of Pharmacy, Medical University of Gdańsk, gen. Hallera 107, 80-416 Gdańsk, Poland; krzysztof.waleron@gumed.edu.pl; 3 Department of Electron Microscopy, Faculty of Biology, University of Gdańsk, Wita Stwosza 59, 80-308 Gdańsk, Poland; magdalena.narajczyk@biol.ug.gda.pl

**Keywords:** POSS conjugate, antibacterial photodynamic inactivation, bacterial infectious disease, drug resistance, phenosafranin photosensitizer, ROS, phenazinum dye, daunorubicin, silsesquioxane, clinical isolates *S. aureus*, *E. coli*, vesicularization

## Abstract

The high photodynamic effect of the Newman strain of the *S. aureus* and of clinical strains of *S. aureus* MRSA 12673 and *E. coli* 12519 are observed for new cationic light-activated phenosafranin polyhedral oligomeric silsesquioxane (POSS) conjugates in vitro. Killing of bacteria was achieved at low concentrations of silsesquioxanes (0.38 µM) after light irradiation (λ_em. max_ = 522 nm, 10.6 mW/cm^2^) for 5 min. Water-soluble POSS-photosensitizers are synthesized by chemically coupling a phenosafranin dye (PSF) (3,7-diamino-5-phenylphenazine chloride) to an inorganic silsesquioxane cage activated by attachment of succinic anhydride rings. The chemical structure of conjugates is confirmed by ^1^H, ^13^C NMR, HRMS, IR, fluorescence spectroscopy and UV-VIS analyzes. The APDI and daunorubicin (DAU) synergy is investigated for POSSPSFDAU conjugates. Confocal microscopy experiments indicate a site of intracellular accumulation of the POSSPSF, whereas iBuPOSSPSF and POSSPSFDAU accumulate in the cell wall or cell membrane. Results from the TEM study show ruptured *S. aureus* cells with leaking cytosolic mass and distorted cells of *E. coli*. Bacterial cells are eradicated by ROS produced upon irradiation of the covalent conjugates that can kill the bacteria by destruction of cellular membranes, intracellular proteins and DNA through the oxidative damage of bacteria.

## 1. Introduction

The rapid acquisition of antibiotic resistance makes it difficult to develop effective methods of eliminating pathogenic bacteria that multiply rapidly under appropriate conditions and can cause infectious diseases on a large scale [1,2,3]. The relentless impact of bacteria on human health makes it necessary to search for new specific nanoparticles (NPs) that can interact with various cellular structures of pathogens [4]. Particular interest may be attributed to situations wherein small molecules are safe for humans and their toxicity increases under conditions of irradiation with light of a specific wavelength [5]. Such conditions are achieved by antimicrobial photodynamic inactivation (APDI), which is also a potential alternative for antibiotics to kill bacteria and importantly, such resistance mechanisms have not yet been observed [6]. APDI employs photosensitizers (PSs) which are excited with visible light to a reactive triplet state and then produce free radicals and other reactive oxygen species (ROS) that are highly toxic to cells [7]. However, most of the phenothiazine, phthalocyanine and porphyrin photosensitizers reported so far are hydrophobic and tend to form aggregates when they interact with bacteria in physiological hydrophilic conditions [8]. Such molecular aggregation and π-π stacking interactions can cause the quenching of the singlet state, thus quenching fluorescence, as well as reducing ROS production and attenuating the effects of both imaging and therapy [9].

The phenosafranin dye is a hydrophilic analog of anthracene in which two carbon atoms in the central aromatic ring are replaced by nitrogen atoms and one of them is substituted with a phenyl group [10]. This positively charged molecule has a wide spectrum of applications in modern research, e.g., as nucleic acid intercalator [11], human ribonucleotide reductase inhibitor [12], protein immobilizer [13] antibacterial agent [14,15,16,17], etc. As a member of the phenazine family, it is a capable of oxidation-reduction reactions and generating ROS, which induces oxidative stress and leads to microbial cell death [18]. It is promising as a next-generation photosensitizer due to its high singlet oxygen (^1^O_2_) quantum yields [19] and good triplet quantum yields (*Φ_T_* = 0.2–0.42) depending on the type of protic solvents [20,21]. They are known to be used as biological probes [22,23], and recently, biotin-phenosafranin conjugate has been described as a photosensitive compound for targeted therapy and imaging [24]. The photodynamic antimicrobial activity of phenosafranin analogs covalently bound to a rigid cage of inorganic polyhedral oligomeric silsesquioxanes (POSSs) has not been studied so far.

POSS cages composed of silicon and oxygen atoms in a 1:1.5 ratio are chemically inert, nontoxic and easy to modify [25,26]. Due to their specific biocompatibility, biodegradability and amphiphilic properties of the siloxane bond structure, they are readily used as drug delivery system [27,28,29]. The small size of the octahedral cage in relation to the nanoparticles of silica, gold nanoparticles, or polymers is extremely beneficial for increasing the transport of biologically active compounds in bacterial cells and penetration through biofilm structures [30]. Additionally, eight-substituted POSS offer the ability of combine several different substituents [31,32]. POSS has been successfully combined with photosensitizers such as porphyrins, chlorin e6, BODIPY as scaffolds for the development of nanoparticles for photodynamic therapy (PDT) [31]. For example, Siano et al. demonstrated the PDT properties of five POSS-porphyrin derivatives for a breast cancer cell line [33]. Zhang et al. modified POSS with porphyrin and found that they completely inhibit aggregation of porphyrin unitsvia stacking [34]. Wang added quaternary ammonium groups to POSS-porphyrin nanoparticles, resulting in an antimicrobial agent with good bacterial membrane intercalation [35]. Recently, a three-dimensional rigid POSS block was introduced into the boron dipyrromethene (BODIPY)-based photosensitizer molecule to alleviate the aggregation of the photosensitizer [36]. Based on the above work, we can conclude that the chemical tenability of POSSs can be used as a promising option to improve the delivery and performance of photosensitizers in photodynamic therapy on bacterial cells.

The main aim of the work is the synthesis of cationic phenosafranin photosensitizers based on POSS cage and the study of antibacterial photodynamic inactivation of gram-negative and gram-positive bacteria. Good solubility and excellent photochemical reactivity of the new organic-inorganic nanoparticles was obtained thanks to the covalent attachment of phenosafranin dye that are positively charged light-activated compounds. According to research, PSF was covalently linked to the inorganic POSS cage via an amide bond on a thioether or siloxane linker, yielding the iBuPOSSPSF, POSSPSF and POSSPSFDAU conjugates, respectively. We used succinic anhydride substituted POSS to achieve high reactivity and provide carboxyl groups and specific siloxane bond structures, which makes the new photosensitizers amphiphilic, increases cellular uptake [37,38] and reduces the toxicity of free PSF in the dark [39,40].

In vitro photodynamic antimicrobial inactivation was investigated with respect to Newman *S. aureus* strain and the clinical methicillin-resistant *S. aureus* (MRSA) 12673 strain as representative of gram-positive bacteria as well as the clinical *E. coli* 12519 strain as representative of gram-negative bacteria. The APDI of the multifunctional light-activated photosensitizer POSSPSFDAU equipped with an amide-bound daunorubicin was compared. The choice of this photosensitizer was due to the encouraging results obtained previously with POSS-daunorubicin conjugates, the cytotoxicity of which has been verified against HeLa eukaryotic neoplastic cells [38]. The in vitro experimental data clearly indicated that these conjugates provide optimal cytotoxicity compared to the free drug and inhibit the viability of HeLa cells. Their advantage is the long-term release of DAU, reducing the local concentration of the anthracycline released and keeping it low level for several days. Combination of an inorganic silsesquioxane cage, hydrophobic DAU and hydrophilic phenosafranin dye in one molecule would significantly improve the solubility of the conjugate in the aqueous environment and facilitate the transport of the antibiotic to the sites where it would intercalate with DNA.

## 2. Results and Discussion

### 2.1. Synthesis and Characterization of Phenosafranin-POSS Photosensitizers

**Figure 1:**
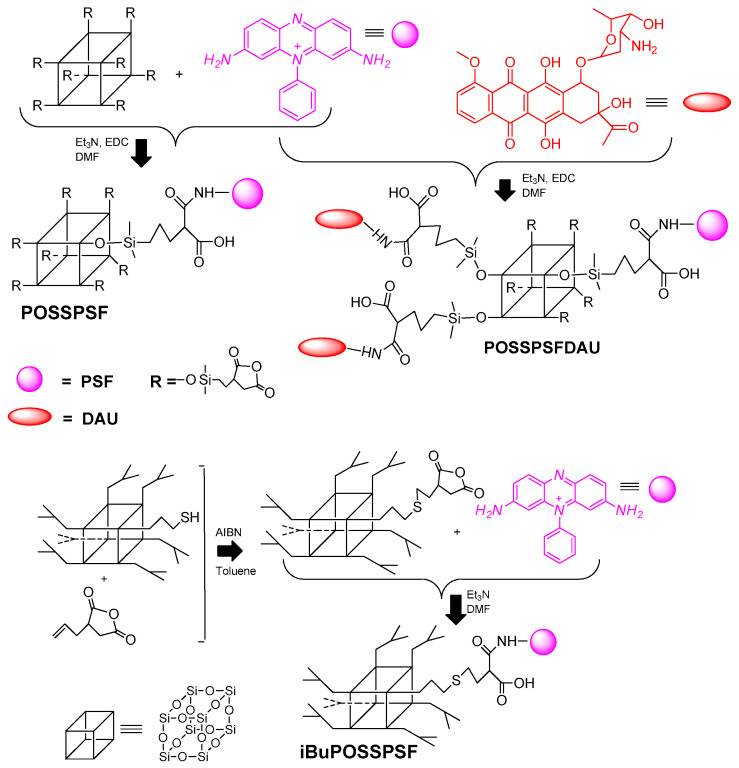
Synthetic route for POSSPSF, POSSPSFDAU and iBuPOSSPSF photosensitizers.

The synthetic pathway for phenosafranin-substituted octasilsesquioxane is shown in Figure 1.

The PSF conjugate, covalently bond to inorganic POSS cage, was successfully synthesized by the reaction of the primary amino group of the dye and 3-[(3-succinic anhydride) propyl)thiopropyl] heptaisobutyloctasilsesquioxane obtained by the efficient thiolene addition reaction [32,39]. We have now shown that the attachment of PSF dye to anhydride substituted POSS via an amide bond on a thioether linker is possible without classical coupling catalysts such as EDC only in the presence of triethylamine in DMF at room temperature. Both PSF and the anthracycline antibiotic daunorubicin (DAU) were conjugated with eight-anhydride substituted POSS through amide bond on a siloxane linker. Reaction of octakis-[3-(2-succinic anhydride)propyl,dimethylsiloxy] octasilsesquioxane (POSSAN) with phenosafranin hydrochloride yields a water-soluble POSSPSF conjugate with an average of two dye molecules per cage as determined by NMR spectroscopy. The photosensitizer covalently bound to DAU was prepared in one-pot synthesis in two successive steps, first with daunorubicin and then with PSF compounds. The final conjugate delivers an average of 1.7 PSF and two DAUs per silsesquioxane cage. Reactions were carried out at room temperature under argon flow and in the dark with DAU. All photosensitizers were pre-purified by precipitation technique and then dialyzed against methanol/water. 

**Figure 2:**
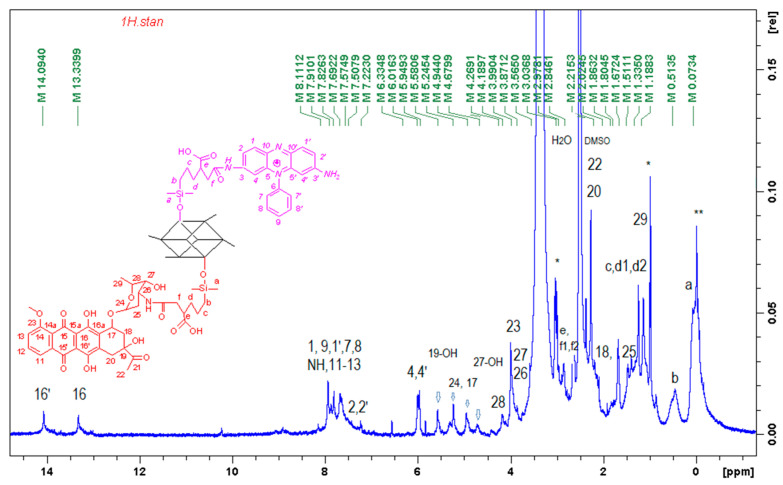
^1^H NMR (500 Hz) spectrum of POSSPSFDAU conjugate in DMSO-d_6_, protons 1 ÷ 10 from PSF and 11 ÷ 29 from DAU (*—trace of Et_3_N, **—trace of vacuum grease).

The chemical structure of the conjugates was verified using ^1^H ^13^C NMR, FTIR and mass spectroscopy and an example spectrum is shown in Figure 2. Although POSSPSFDAU nanoparticles have a rather complicated ^1^H NMR spectrum, the characteristic resonance lines for PSF, antibiotic and POSS molecules are found in three non-overlapping regions, making it easier to assign resonance lines to the correct protons [41,42]. Thus, POSS-cage protons occupy a high field area of 0–3 ppm, most of the daunorubicin protons (apart from the C16-OH and C16′-OH protons at 13.34 and 14.09 ppm) occupy the middle of the spectrum 3.5–5.6 ppm and PSF occupies the aromatic area from 6 to 8.5 ppm with nitrogen about 7.73 ppm (Figure 2). The new amide bond confirms a proton shift of the daunorubicin C-26 sugar moiety from 3.3 ppm to 3.87 ppm. 2D NMR spectra such as H-H cosy, H-C HSQC and HMBC were run in parallel to confirm the presence of the expected functional groups. Detailed chemical shifts of proton and carbon signals for the POSSPSF, POSSPSFDAU and iBuPOSSPSF photosensitizers are presented in Table 1.

**Figure 3:**
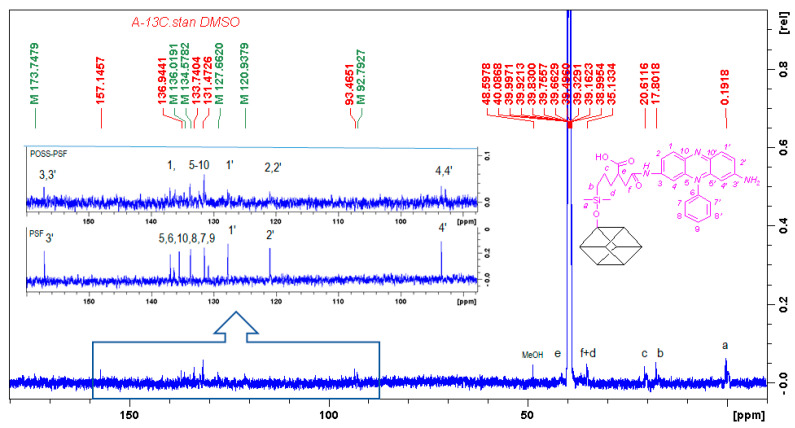
Comparison of the ^13^C NMR (500 Hz) spectra of POSSPSF conjugate (bottom), expansion of the 160–85 ppm range (top) and PSF (in the middle) in DMSO-d6.

The covalent attachment of PSF molecule faintly shows the ^13^C spectrum (Figure 3). The shifts of nitrogen-bonded carbon atoms before and after the grafting overlap at 157.1 ppm (major mono-substituted PSF conjugates are formed). On the other hand, there is a significant shift of the C4‘and C4 carbons from 92.7 to 93.5 ppm, as well as the C1’ and C1 carbons, which are shifted from 127.7 to 136.9 ppm. The remaining PSF carbon atoms are seen in the range of 130–136 ppm. The alkyl link in the POSS cage is labeled at 0.4, 17.8, 20.6, 35.1 and 40.1 ppm for Ca, Cb, Cc, Cd, Ce and Cf, respectively, while a small signal at 173.7 ppm describes carbonyl group.

**Figure 4:**
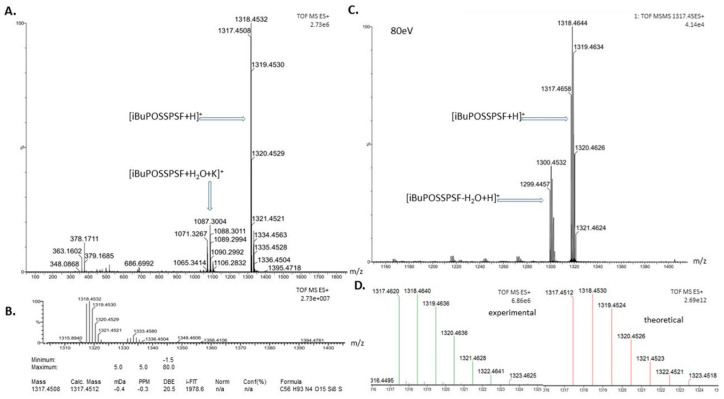
Analysis of the iBuPOSS-PSF conjugate by an electrospray source in positive mode by mass spectrometer with TOF analyzer in (**A**); ESI (+)—HRMS analysis in (**B**); spectrum of iBuPOSSPSF fragmentation experiments (*m*/*z* 1317.45), collision energy was 80 eV in (**C**); simulated spectrum [C_56_H_94_N_4_O_15_Si_8_S + H] + (red line) and experimental (green lines) in (**D**).

High resolution mass spectroscopy (HRMS) has been used as a tool to identify photosensitizers by determining the exact mass, isotope distribution and fragmentation pathway. We experimentally tested various methods such as electrospray (ESI) in negative mode [43] and atmospheric pressure photoionization (APPI) in positive mode [39] for POSS substituted with daunorubicin and phenosafranin dye, respectively. In this line, we show that according to the chemical functions of iBuPOSSPSF conjugate, the positive mode EI-ToF-MS was the most suitable method for structure confirmation as shown in Figure 4. Thus, we identified a molecular ion at *m*/*z* 1317.4508 relative to [(POSS-(iBu)_7_)(PSF) + H]^+^ structure, as well as two ions *m*/*z* 1087.3026 and 1071.3267 with respect to the open anhydride-POSS. Additionally, the low intensity peak can be attributed to the protonated iBuPOSS-PSF molecule devoid of one water molecule, indicating the assistance of phenosafranine amino groups in the opening of the cage after ESI [44]. After protonation of the primary amine group of PSF, it is possible to transfer this proton to the oxygen in the cage, inducing the cleavage of a Si-O covalent bond and leading to the formation of a hydroxyl group and a positively charged Si atom. The amine group could then attack this charged Si atom in the backbiting process, yielding a ring from which the proton would be transferred to the oxygen atom of the OH group, allowing the water molecule to be expelled and the *m*/*z* 1299.4457 product ion to be formed.

### 2.2. UV Absorption and Fluorescence Spectroscopic Studies

**Figure 5:**
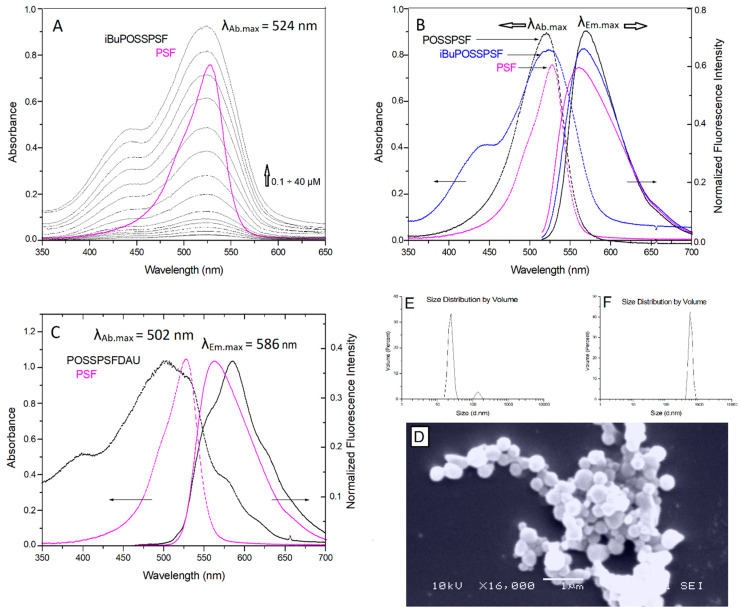
UV-absorption spectra measured for iBuPOSSPSF in the concentration range from 0.1 µM to 40 µM) in methanol (black lines) and for PSF dye (magenta line) (λ_Ab.max_ = 529 nm) in (**A**); normalized absorption (dashed line) and emission (solid line) spectra of iBuPOSSPSF (λ_Ab.max_ = 524 nm, λ_Em.max_ = 567 nm), POSSPSF conjugates (λ_Ab.max_ = 520 nm, λ_Em.max_ = 570 nm) and PSF dye in (**B**); normalized absorption—emission spectra of POSSPSFDAU conjugate (λ_Ab.max_ = 502 nm, λ_Em.max_ = 586 nm) in (**C**); SEM micrograph of POSSPSFDAU nanostructures in (**D**); hydrodynamic diameter determined by DLS measurements in methanol (d = 24 nm SD ± 3.5 nm) in (**E**) and in water (d = 560 nm SD ± 89 nm) in (**F**). Emission spectra were excited in methanol at a wavelength of 500 and 480 nm, slit 3.

The UV-visible and fluorescence studies reveal different spectroscopic responses of PSF following binding interactions with iBuPOSS, succinic anhydride-POSS and daunorubicin-POSS. The significant observation of this study is: PSF dye displaying a broad unstructured absorption band with a maximum at 529 nm in methanol [24]. Due to the combination of a silsesquioxane cage, the absorption profile of PSF undergoes a slight hypsochromic shift of their maximum absorbance. We observe a shift of 5 nm, 9 nm and 27 nm for isobutyl, anhydride and daunorubicin, respectively, in the direction of the shorter wavelength, as in Figure 5. The blue shift of the PSF-conjugate absorption peaks suggests an increase in polarity in the vicinity of the dye molecules conjugated with silsesquioxane cage compared to bare dye [13]. The low band at 440 nm for iBuPOSSPSF indicates the asymmetric structure of the conjugate, which is composed of a planar phenazine system and a spherical silsesquioxane cage [45]. The significant shift in the absorption profile of POSSPSFDAU is partly due to the optical properties of daunorubicin and partly to the binding environment (Figure 5C). As previously reported, partial polarization in the POSS-PSF molecule leads to electron transfer between atoms and influences different interaction behavior with solvents compared to the free PSF dye known as the solvatochromic effect [20].

The fluorescence emission spectra of the conjugates in methanol solutions excited at the wavelength, λ_e__x_ = 500 nm confirms the grafting of the cationic dye into the inorganic cage, which is manifested by the shift of the maximum fluorescence wavelength to 567 nm, 570 nm and 586 nm relative to phenosafranin bare (λ_em_ = 560 nm) (Figure 5B,C). This bathochrome shift suggests an interaction of the dye binding to the cage and an increase in polarity in the vicinity of the dye. Subsequently, these differences may play a significant role in the interaction of the conjugates with the bacterial cell wall. This also found to be in good harmony with the literature reports [46].

### 2.3. Shape and Morphology

Modification of POSS corners with PSF dye leads to fluorescent nanodots that are good candidates for antibacterial photodynamic therapy. The bulky steric hindrance resulting from PSF moieties located at the flanking arms of the octahedral silsesquioxane scaffold ensures the good optical activity and stability of the conjugate in methanol and PBS buffer [39]. The POSSPSFDAU dynamic light scattering measurements revealed the main population of nanoparticles with an average hydrodynamic diameter of 24 nm and narrow size distribution in methanol (Figure 5E). However in water, the same photosensitizer reveals much larger aggregates with a diameter of up to 560 nm as a result of the interaction of the hydrogen bonds of the positively charged molecule (Figure 5F). This is confirmed by scanning electron microscopy (SEM) images, where the conjugate forms regular spherical NPs with a diameter of 200–600 nm (Figure 5D). Therefore, we expect that in the environment of the bacterial cell wall, rich in lipopolysaccharides, peptidoglycan and proteins, the tendency to aggregate division and better penetration of bacteria will increase.

### 2.4. Photoinactivation of Gram-Positive and Gram-Negative Bacteria In Vitro

Three new phenosafranin derivatives have been successfully tested as potential photosensitizers in antibacterial photodynamic therapy. The bacterial suspensions were combined with conjugate solutions in PBS buffer, then incubated for 15, 30, 60 and 120 min at 37 °C then irradiated with green light (λ_em. max_ = 522 nm, 10.6 mW/cm^2^) for 5 min. The killing efficiency of POSSPSF, iBuPOSSPSF and POSSPSFDAU against bacteria at concentration of 0.38 µM and 6.3 µM was subsequently determined. Since bacterial killing efficiency is the result of a combination of photosensitizer type and light energy as well as binding to the bacterial cell and intracellular accumulation [47], we chose gram-positive and gram-negative bacteria represented by Newman and clinical MRSA 12673 *S. aureus* and *E. coli* 12519 strains for the study.

**Figure 6:**
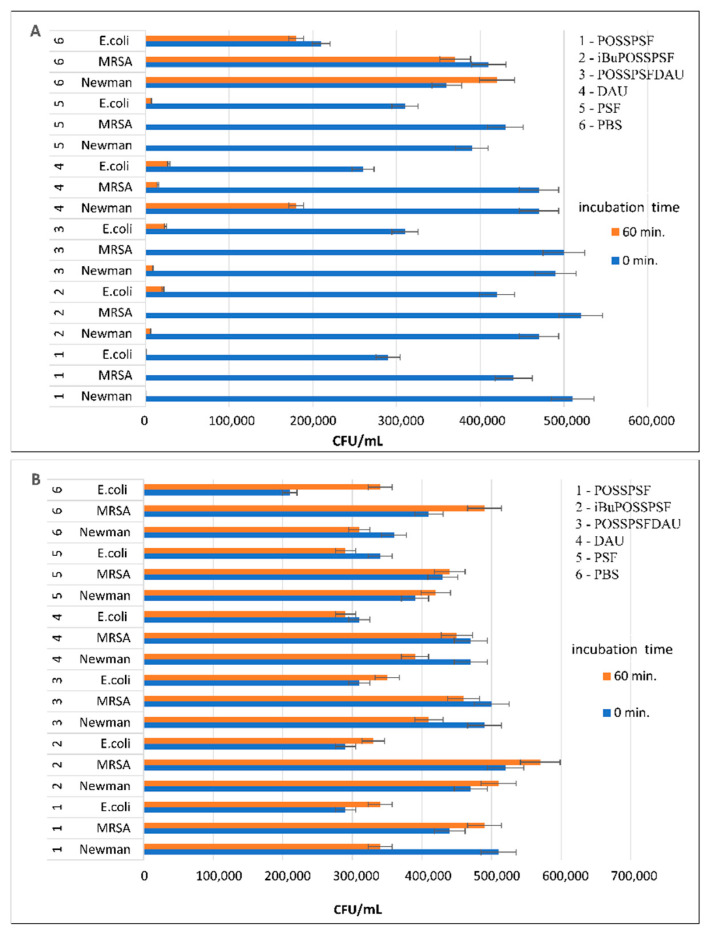
Photodynamic inactivation of POSSPSF, iBuPOSSPSF and POSSPSFDAU against *S. aureus* strains Newman, MRSA and *E. coli.* The bacterial suspensions were incubated with photosensitizer at concentration 0.38 μM for 60 min at 37 °C followed with green light irradiation (λ_em. max_ = 522 nm, irradiation = 10.6 mW/cm^2^) for 5 min (**A**) and without irradiation (**B**).

No dark cytotoxicity was observed in all experiments with the selected bacteria as shown in Figure 6B. The lowest concentration of POSSPSF (0.38 µM) caused complete eradication of the *S. aureus* and *E. coli* bacterial cells after irradiation for 5 min. Both iBuPOSS and POSSPSFDAU also destroy all cells of the drug-resistant MRSA strain under the same conditions. In addition, iBuPOSSPSF reduces 98% of *S. aureus* Newman cells and 95% of *E. coli* cells. Moreover, the POSSPSFDAU photosensitizer kills 100% of MRSA cells and 97% of Newman cells, i.e., the conjugate kills bacteria more easily and more effectively than free daunorubicin (Figure 6A). The photodynamic antibacterial effect of the phenosafranin-POSS derivatives does not exceed that of free PSF. However, this is an observation for only two strains of *S. aureus* and *E. coli.* Extending the research to other pathogens may lead to the discovery of the high selectivity of POSS-PSF nanoparticles. On the other hand, the value of the photosensitizer should not be measured only by the fire power, but also the biodegradation pathway, toxicity to eucaryotic cells and environmental impact are of great importance. POSS cage conjugation offers the prospect of a cellular uptake, a biocompatible carrier with a human and environmentally friendly biodegradation pathway. However, they are not tested in this manuscript. We only carried out preliminary studies which show that POSS-photosensitizers are stable and provide high photodynamic efficiency in eradication of bacteria and lead to bacteria cell death at low photosensitizer concentration.

**Figure 7:**
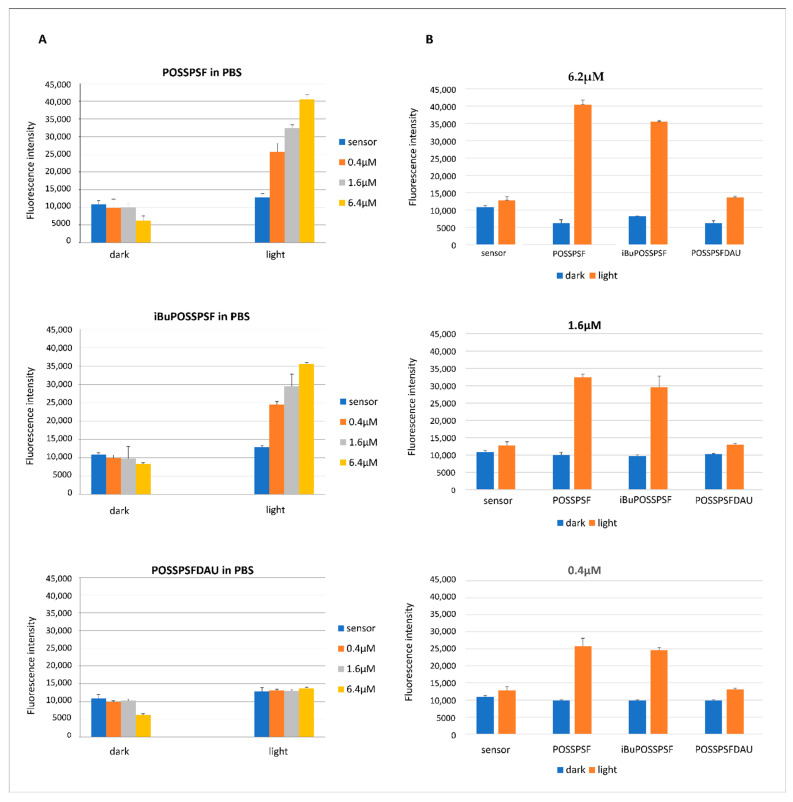
Singlet oxygen detection. Comparison of POSSPSF, iBuPOSSPSF and POSSPSFDAU compounds in PBS buffer (pH = 7.4) at three concentrations (0.4, 1.6 and 6.2 µM) in the presence of a sensor (SOSG), under light (λ_em. max_ = 522 nm, 10.6 mW/cm^2^) and dark conditions (**A**) and analysis of all conjugates considered at particular concentrations, exposed to light and dark (**B**).

The singlet oxygen [^1^O_2_] detection was carried out to ascertain whether singlet oxygen generation correlated with the observed bactericidal activity of the conjugates. The singlet oxygen sensor green probe (SOSG) was used to test whether conjugates in a PBS buffer environment after irradiation with green light (λ_em. max_ = 522 nm, 10.6 mW/cm^2^) for 5 min could lead to the generation of singlet oxygen. The results presented in Figure 7 showed that the concentration of singlet oxygen produced (represented by the highest fluorescence level) from the POSSPSF and iBuPOSSPSF photosensitizers during irradiation with green light was very high. Whereas the POSSPSFDAU showed the lowest increase in fluorescence. These studies support our hypothesis that bacterial inactivation may be caused by the generation of reactive oxygen species (ROS).

**Figure 8:**
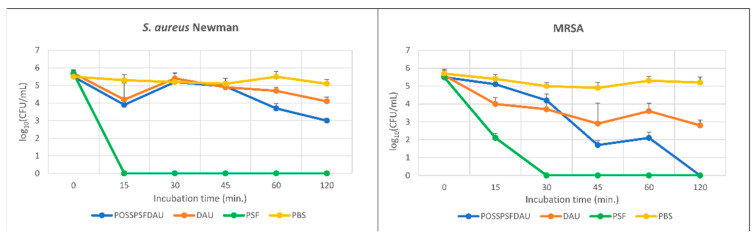
Photodynamic inactivation of MRSA and Newman *S. aureus* in vitro by POSSPSFDAU conjugate, DAU and PSF dye after irradiation for 5 min (λ_em. max_ = 522 nm, irradiation = 10.6 mW/cm^2^).

The antibacterial effect of POSSPSFDAU increases with the incubation time that may indicate on the slow release of the drug, achieving nearly 100% and 100% cell eradication of bacterial cells after 60- and 120-min incubation, respectively (Figure 8). Based on the experiments performed, it is difficult to say whether the antibacterial activity of this multifunctional conjugate is due to photodynamic inactivation or the release of the drug daunorubicin or both. Most likely, both mechanisms contribute to the better activity of conjugate compared to DAU. The POSSPSFDAU conjugate appears to be a promising compound with potential antibacterial activity, considering that this conjugate showed much lower cytotoxicity than free DAU, especially at concentrations active against bacterial cells [38]. Daunorubicin is an anthracycline antibiotic with antineoplastic activity, used in the therapy of acute leukemia and AIDS related Kaposi sarcoma. Daunorubicin is cytotoxic by interacting with DNA mediated by topoisomerase, thereby inhibiting DNA replication. Antimicrobial activity for this group of antibiotics has also been documented against gram-positive bacteria [48]. It was reported that the anthraquinone chromophore of the daunorubicin molecule generates a triplet state, but the quantum yield of singlet oxygen is extremely low (*Φ_T_* = 0.02–0.03), suggesting that anthracyclines are weak photodynamic sensitizers. [49] However, for this reason, we observe inactivation of *E. coli* and *S. aureus* incubated with daunorubicin, followed by irradiation with green light. *E. coli* has also been shown to be sensitive to high molecular weight antibiotics of the anthracycline groups, demonstrating a strong synergistic effect with rifampicin against *E. coli* and *Acinetobacter baumannii* strains [50].

In conclusion, these cationic PSF-silsesquioxane nanoparticles show high APDT activity as a result of ROS generation typical of the phenosafranin dye [19]. Moreover, they integrate perfectly with both gram-positive and gram-negative bacteria, which is in line with the Anderson observations [51,52]. Both monoderm and diderm bacterium types have an overall negatively charged cell surface consisting of different surface structures [53]. The cell surface of the diderm *E. coli* has a smaller negative charge than the one of the monoderm *S. aureus* that is rich in peptidoglycan [54]. The anionic surface thus acts as an electroattractive scaffold for cationic photosensitizers, which are more efficiently bound to and taken up by bacteria [55].

### 2.5. Antibacterial Mechanism of POSS-Photosensitizers

**Figure 9:**
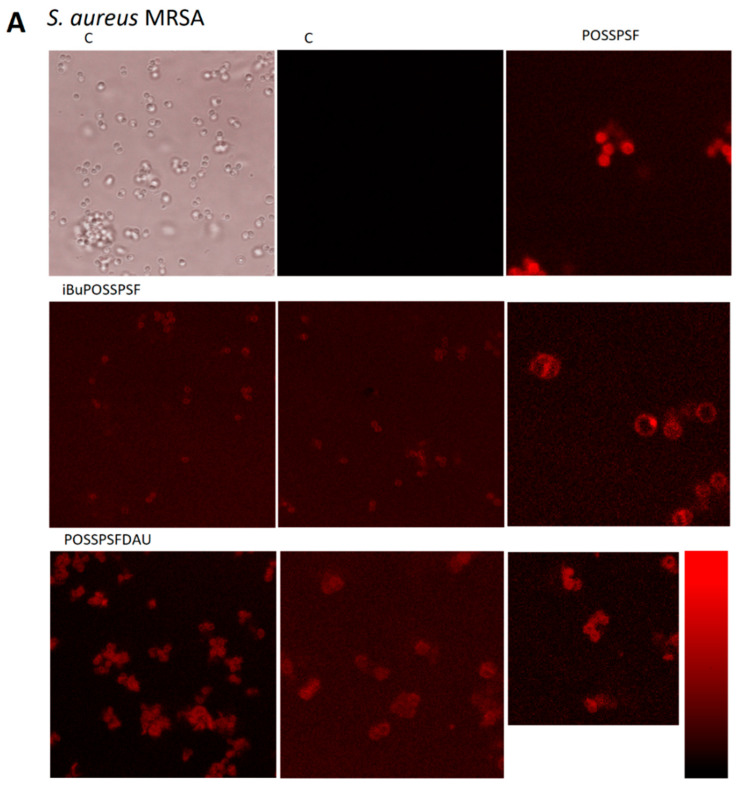

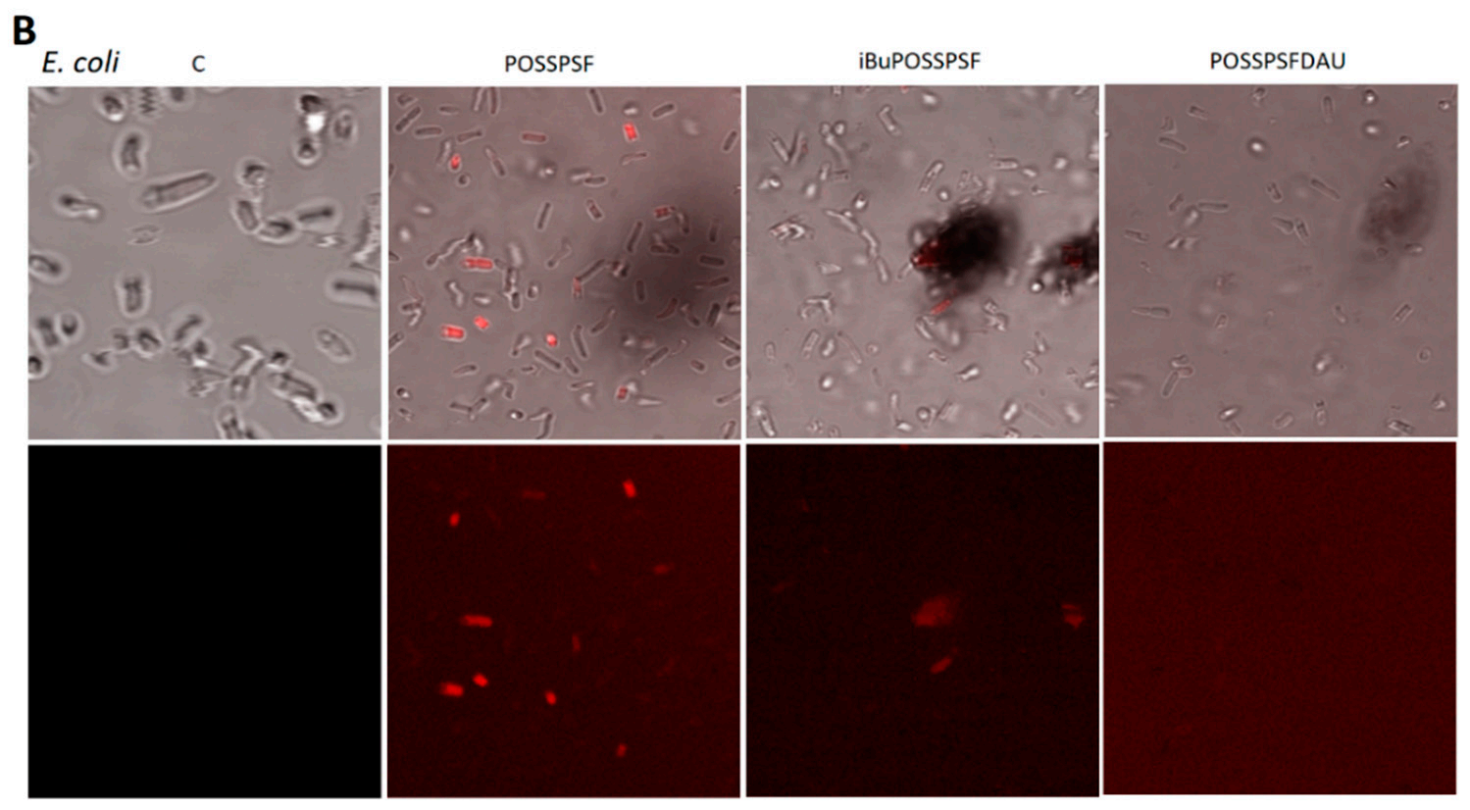
Confocal microscopy images of *Staphylococcus aureus* strain MRSA 12673 (**A**) and *Escherichia coli* 12519 (**B**) in the absence (control, c) and presence of POSSPSF, iBuPOSSPSF and POSSPSFDAU.

Almost complete eradication of MRSA and the Newman *S. aureus* and *E. coli* strains suggests that the positive charge and hydrophobic interaction of alkyl linkers and siloxane bonds in POSS-photosensitizers may synergistically promote the anchoring of nanoparticles to the bacterial outer membrane/cell wall or accumulation inside of bacteria [53]. The POSSPSF conjugate forms the smallest nanoparticles (average size 8 nm according to DLS) where the POSS domains is the core of nano-micelle, whereas positively charged PSF corona plays an important role in preventing intermicellar aggregation in water [56]. These nanoparticles enter the cell of both *S. aureus* and *E. coli*. Slightly larger nanoparticles of the multifunctional conjugate POSSPSFDAU poorly penetrate the bacterial wall, or rather anchor in it, as shown by confocal microscopy. Furthermore, iBuPOSSPSF accumulates in the *S. aureus* cell membrane/cell wall, indicating that the morphology and amphiphilicity of the photosensitizer are probably the most important factors in determining the effectiveness of the photosensitizer (Figure 9).

This is probably the reason why POSSPSF penetrates *S. aureus* and *E. coli* more easily, while iBuPOSSPSF and POSSPSFDAU accumulate in the *S. aureus* cell wall or cell membrane (in both cases, no fluorescence was observed inside the bacteria cell) (Figure 9A). It is difficult to say whether the conjugates accumulate between multiple layers of peptidoglycan, get stuck in the pores of the peptidoglycan, interact with NAM and NAG murein units, or with negatively charged acid groups anchored to murein or accumulate within cell membrane, but numerous damaged and aggregated *S. aureus* cells are visible.

It appears that the most important factor determining the mode of action and antimicrobial activity is the hydrophilic-hydrophobic nature of the conjugates. Thus, POSSPSF contains, in addition to siloxane bonds and a positively charged PSF group and depending on the pH, carbonyl and carboxyl groups, which increases the solubility of nanoparticles in the aqueous environment and increases cellular uptake [37]. Modification of the photosensitizer with hydrophobic daunorubicin reduces the solubility of the POSSPSFDAU conjugate and reduces the affinity binding to the bacterial cell wall (reduces the migration of nanoparticles through ion channels in the cell membrane). Similarly, organic butyl groups reduce the amphiphilicity of nanoparticles. The numerous hydrophobic domains present in both photosensitizers predispose them to accumulate in the peptidoglycan layer of the *S. aureus* cell wall.

In the case of *E. coli*, indeed, the additional outer membrane, which is an integral element of the cell wall, may constitute an additional barrier to photosensitizers. However, the confocal microscope images (Figure 9B) also show numerous, non-luminous, damaged *E. coli* cells, which may indicate that these compounds affect the cells, but are removed from them. We hypothesize that the lower antibacterial activity against *E. coli* observed for tested compounds are due to the active transport outside (efflux pumps or vesicularization) of the bacteria cells or simply because of leakage of material from the cell. However, the vesicularization is visible on the TEM images (Supplementary Materials, Figure S1B,C). Literature data suggest that efflux pumps as well as membrane vesicles (MVs) may be used by the cell as a first-line defense mechanism, preventing the drug from reaching lethal concentrations [57,58,59].

**Figure 10:**
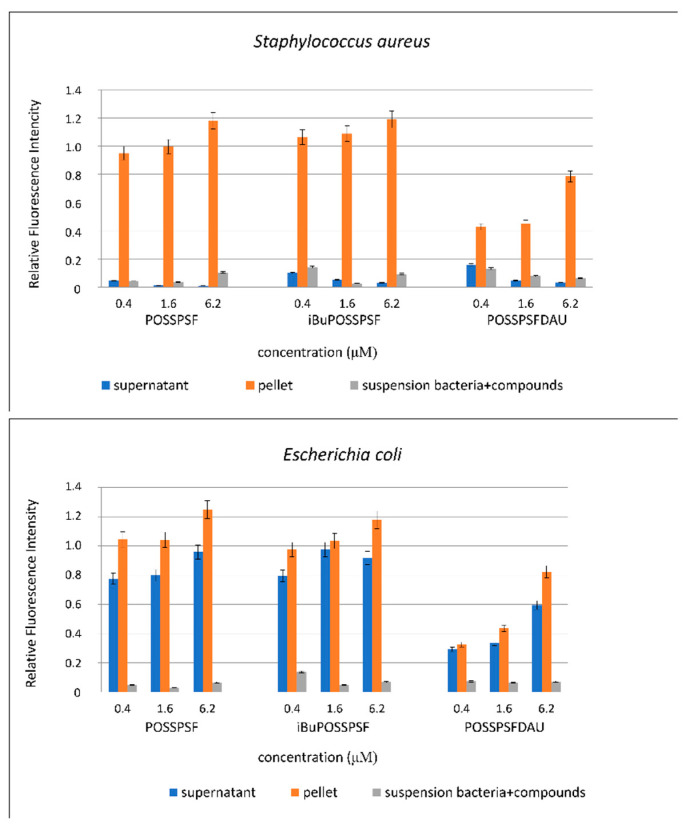
Fluorescence intensity of *S. aureus* and *E. coli* cells suspension, pellet and supernatant incubated in the presence of different concentration of POSSPSF, iBuPOSSPSF and POSSPSFDAU. Relative fluorescence intensity was calculated to the fluorescence of photosensitizers solution.

The above considerations are confirmed by the results of experiments, where the intensity of fluorescence in the pellet and in the supernatant was analyzed after centrifuging a suspension of *S. aureus* and *E. coli*, incubated in the presence of conjugates—POSSPSF, iBuPOSSPSF and POSSPSFDAU. The results of these experiments are shown in Figure 10. The fluorescence of the initial solutions for both strain samples is the same—the graphs almost match. In case of POSSPSF, the fluorescence intensity of the cells in the pellet for both bacteria are similar, suggesting that the conjugate was absorbed into both bacteria in a similar amount. However, significant differences in fluorescence were observed with the supernatants. The supernatant obtained by centrifuging the mixture of *S. aureus* cells and POSSPSF showed very low fluorescence intensity, while the fluorescence of *E. coli* supernatant was comparable to that of pellet. The POSSPSF conjugate binds strongly to the *S. aureus* cell (penetrates inside) so that it cannot be removed. The results also indicated that as the concentration of conjugate in the initial suspension increases, the concentration of POSSPSF in the supernatant decreases, which might suggest that any amount of POSSPSF is being absorbed by *S. aureus*. In order to eliminate large disproportions between the fluorescence intensity of the sediment and the supernatant, we conducted an additional experiment. Namely, after centrifugation from a 2 mL of bacterial suspension with photosensitizer, the bacteria were suspended in the 2 mL of BPS buffer and 200 μL of bacteria suspension was collected and centrifuged. Then, 200 μL of supernatant and 200 μL of resuspended pellet was used for measurements. The results are presented as graphs with relative fluorescence intensity calculated to the fluorescence of photosensitizers solutions (Figure S9, Supplementary Materials). On the basis of this graph, it can be concluded that the experiment carried out in this way eliminates the large disproportion between the fluorescence intensity of the sediment and the supernatant. However, the fluorescence intensity of the sediment is still much higher than that of the supernatant, which may suggest absorption of the compound by *S. aureus* cells. On the other hand, we do not exclude, in this case, that low fluorescence intensity of POSSPSF supernatant is related to the phenomenon of aggregations or precipitation in the presence of *S. aureus* cells or compounds produced by the bacteria. This phenomenon, however, requires a detailed explanation in further experiments.

In the case of *E. coli*, even at the lowest concentration, the content of POSSPSF in the pellet and supernatant is high. This suggests that the POSSPSF conjugate is equally eager to penetrate of *E. coli* cells and is equally eager to be removed from it, or one part of it penetrates and other part stays outside the bacterium. Similar results were obtained for the other two conjugates, iBuPOSSPSF and POSSPSFDAU. The only difference in this case concerns the much lower fluorescence intensity of the pellets of both bacteria, 4- and 8-times lower fluorescence was observed for iBuPOSSPSF and POSSPSFDAU, respectively, compared to POSSPSF. 

The results described above indicate a completely different mechanism of action of the conjugates against *S. aureus* and *E. coli* cells. We hypothesize that the high intensity of supernatant fluorescence in the case of *E. coli* cells may be related to the phenomenon of vesicularization, i.e., the production of membrane vesicles. Membrane vesicles (MVs) are produced through controlled blebbing of the outer-membrane of the gram-negative bacteria (OV_S_) and can also be formed by endolysin-triggered cell lysis. Recent work has shown that vesicles can be also produced by gram-positive bacteria [57]. In figures containing *S. aureus,* cells such vesicles were not observed. However, these structures are observed in *E. coli* cell samples incubated in the presence of POSSPSF and iBuPOSSPSF (TEM, Supplementary Materials, Figure S1). In the case of POSSPSFDAU, the presence of the above-mentioned vesicles was not noticed, which suggests a different type of interaction. We suppose that the high fluorescence intensity of supernatant may be due to the active transport of POSSPSFDAU outside the cell or simply leakage of material from the cell. Indeed, images of *E. coli* pellet cells from a confocal microscope (Figure 9B) show no fluorescence. In the scientific literature, where the derivatives of antibiotics from the anthracycline group were used, it has been shown that the resistance of *E. coli* bacteria results from the active transport of compounds out of the cell. For the research the efflux deficient delta *tolC E. coli* BW25113 strain were used [50].

**Figure 11:**
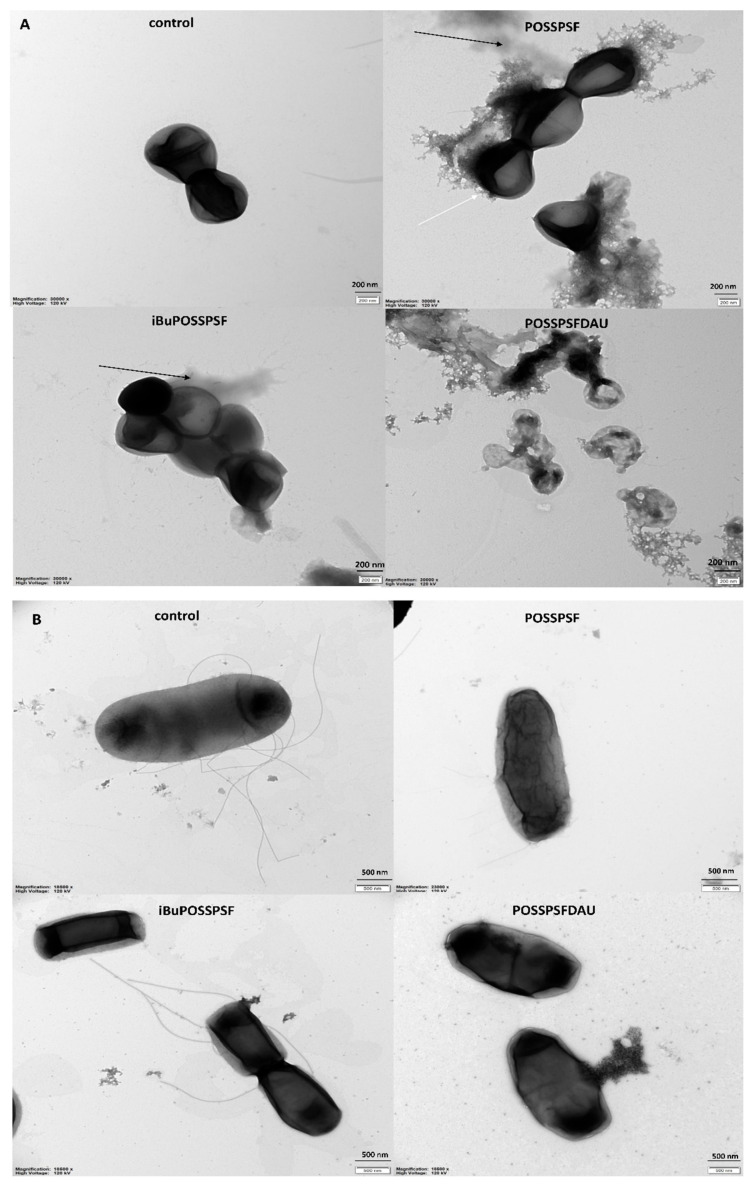
Transmission electron microscopy images of *Staphylococcus aureus* strain MRSA 12673 (**A**) and *Escherichia coli* 12519 (**B**) in the absence (control) and presence of POSSPSF, iBuPOSSPSF and POSSPSFDAU (green light irradiation (λ_em. max_ = 522 nm, irradiation = 10.6 mW/cm^2^) for 5 min).

To visualize the effect of POSSPSF, iBuPOSSPSF and POSSPSFDAU on bacterial cell, transmission electron microscopy was also carried out (Figure 11). For these tests, *S. aureus* MRSA 12673 and *E. coli* 12519 cells were selected. The images of the bacterial cells treated with tested compounds showed distortion in the shape and morphology, while the regular shape of the control cells was clearly visible. Some cells turned from the normal round (Figure 11A) or rod-shaped (Figure 11B) into irregular shapes presenting broken and lysed cells with the cellular mass leaking out (*S. aureus* strain MRSA), black arrows) (Figure 11A). The *E. coli* cells have become distorted and thickenings are visible in some places (Figure 11B). The cells coated with tested compounds are also visible (indicated by white arrow). Changes in bacterial cell morphology can indicate that cell membrane permeability was disrupted or/and the structure of the cell wall was disintegrated (more figures are introduced to the Supplementary Materials, Figure S2).

## 3. Materials and Methods

### 3.1. Materials

Octakis-[3-(2-succinic anhydride)propyl, dimethylsiloxy] octasilsesquioxane (POSSAN) was obtained by hydrosililation of allyl succinic anhydride with octakis-(hydridodimethylsiloxy) octasilsesquioxane by the method described previously [43]. Phenosafranin hydrochloride (3,7-diamino- 5-phenylphenazinum chloride), (PSF), allyl succinic anhydride, 2,2′-azobisisobutyronitryl (AIBN), triethylamine, N-(3-dimethylaminopropyl)-N′- ethylcarbodiimide (EDC), methanol and dimethylformamide (DMF) were of analytical grade and were supplied by Sigma-Aldrich (Saint Louis, MO, USA). 3-Mercaptopropyl-heptaisobutyl octasilsesquioxane (iBuPOSS) from Hybrid Plastics (Hattiesburg, MS, USA,) and daunorubicin hydrochloride (DAU) from Beijing Packbuy M&C company (Beijing, China) and were used without purification. The dialysis bag (MWCO, 1000) and Spectra-Por Float-A-Lyzer G2 were purchased from Sigma-Aldrich (Saint Louis, MO, USA). All reactions were carried out in argon atmosphere in Schlenk flasks. PBS (phosphate-buffered saline, pH = 7.4) for microbiological studies was used. The bacterial strains used in the study were obtained from the Intercollegiate Faculty of Biotechnology, University of Gdansk and Medical University of Gdansk collection (*Staphylococcus aureus* strain Newman), and from the Department of Pharmaceutical Microbiology, Medical University of Gdansk collection (clinical strains of *Staphylococcus aureus* MRSA 12673 and *Escherichia coli* 12519). Strains were stored as glycerol stocks at −70 °C. For research purposes, cultures were conducted at 37 °C for 18 h in Brain Heart Infusion Broth (BHI) (Biomaxima S.A., Lublin, Poland).

### 3.2. Measurements

The structures of POSSPSF, iBuPOSSPSF and POSSPSFDAU conjugates were identified on the basis of ^1^H and ^13^C NMR spectroscopy applying COSY, HMBC, HSQC techniques. The NMR spectra were run on Bruker AVANCE III DRX-500 MHz spectrometer equipped with 5 mm inverse broadband dual channel probe head with Z-gradients, operating at 500.13 and 125.77 MHz for ^1^H and ^13^C, respectively. All measurements were carried out at 295 K and the temperature was stabilized with Bruker BCU 05 cooling system controlled by VTU 3200 unit. Spectra were acquired and processed with TopSpin 3.6 Bruker software running under Windows 10 platform. For all spectra, the chemical shift values were referenced externally to TMS (d = 0.00 ppm for ^1^H and ^13^C). 

High resolution mass spectrometry (HRMS) analyses were run using Synapt G2-Si mass spectrometer (Waters) equipped with a quadrupole-time-of-flight mass analyzer. Methanol was used as a solvent. The measurements were performed in positive ion mode with the desolvation gas flow at 500 L/h and capillary voltage set to 2500 V with the flow rate 100 L/min. The results of the measurements were processed using the MassLynkx: 4.1 software (Waters) incorporated with the instrument. 

Matrix-assisted laser desorption ionization time of flight (MALDI-TOF) measurements were performed using a Voyager Elite (PerSeptive Biosystems, Framingham, MA, USA) instrument equipped with a pulsed N2 laser operating at 337 nm. Mass spectra were obtained in the linear mode with an accelerating voltage of 20 kV. Samples were prepared from THF solutions with DT, 1,8-dihydroxy-9(10H)-antracenone (dithranol) as a matrix and potassium trifluoroacetate (AgTFA) as a cationizing agent.

Attenuated Total Reflectance (ATR) FT-IR spectra of the conjugates were measured on a Thermo Scientific Nicolet 6700 FT-IR instrument (Golden Gate ATR). Spectra were recorded averaging 64 interferograms with a resolution of 2 cm^−1^. The measurements were repeated for three independent samples of powder.

The size and morphology of POSS-photosensitizers in methanol and aqueous solution were measured by scanning electron microscopy (SEM, 10 kV, Jeol, JSM-5500LV). The average hydrodynamic particle size and polydispersity index were investigated by Zetasizer 3000 HS instrument (Malvern, UK) equipped with a 5 mW Helium-Neon ion laser operating at λ = 632 nm. 

The UV-vis absorption spectra were recorded by Specord S600 model diode-array spectrometer (Analytic Jena, Jena, Germany). The optical path length was 1 cm. The fluorescence spectra were measured in quartz cuvettes (10 mm) using a Horiba (Jobin Yvon) fluorimeter. The widths of the excitation and emission slits were set to 5.0 or 3 nm. The excitation wavelength for fluorescence spectra measurements was 500 nm for POSSPSF, iBuPOSSPSF and 480 nm for POSSPSFDAU, respectively.

### 3.3. Synthesis of the Cationic Phenosafranin-POSS Photosensitizers

Phenosafranin-inorganic cage conjugate, POSSPSF was synthesized by reacting the primary amino groups of PSF and octakis-[3-(2-succinic anhydride)propyl, dimethylsiloxy] octasilsesquioxane (POSSAN) in solution (DMF) in the presence of excess of trimethylamine and EDC, as previously described [39]. The average number of PSF calculated from the ^1^H NMR spectrum of the POSSPSF conjugate was two dyes per POSS-cube and detailed NMR data are shown in Table 1. Additionally, *m*/*z* (MALDI-TOF MS) found: [M + H]+ 2421.8, molecular formula C_90_H_136_N_4_O_44_Si_16_ requires 2423.48, λ_absorb.max_ = 520 nm in methanol.

POSSPSFDAU conjugate bearing both daunorubicin and light-activated phenosafranin dye was obtain in one-pot synthesis. Before reaction, daunorubicin hydrochloride, (DAU) (237 mg, 0.42 mmol) was dissolved in dry DMF (10 mL) and mixed with triethylamine (170 mg, 1.68 mmol) to neutralize the ammonium sites on the DAU units. Initially, POSSAN (300 mg, 0.14 mmol) was dissolved in 5 mL anhydrous DMF and added to the DAU solution, followed by agitation for 2 h at room temperature in the dark to attach the antracycline. Then, the phenosafranin hydrochloride (135 mg, 0.42 mmol) in 10 mL of dry DMF and EDC (154 mg, 0.80 mmol) was added (feed molar ratio 1:3:3 mol/mol, POSSAN: PSF: DAU) and continued stirring 48 h to complete the reaction. Progress of the reaction was followed by TLC method using MeOH/H_2_O/AcOEt (2:1:1) as mobile phase. The solvent and unreacted amine was evaporated in reduced pressure and the resulting mixture was dissolved in methanol and precipitated in hexane. The dark red precipitate was dried in a vacuum (yield 78%) and successively dialyzed (MWCO 1kD) against methanol for 24 h and water for 12 h. Finally, the POSSPSFDAU dry powder containing on average of 1.7 PSF and two DAU molecules per cage was obtained after lyophilization of the above dialyzed solution and analyzed by ^1^H and ^13^C NMR spectroscopy, data are collected in Table 1. FTIR (cm^−1^): 3360 ν (O-H and N-H); 2952 ν (C-H); 1708 ν (C=O); 1651 ν (C=O, amide I); 1635 ν (C=C, aromatic ring PSF); 1576 ν (C=C, aromatic ring, DAU); 1532 ν (C-C, C-H); 1491 (C=C, aromatic ring PSF); 1458 δ (-NH, secondary amine); 1407 δ (O-C-H, N-C-H, DAU); 1349 ν (C-N, secondary amine); 1286 δ (C-O-H, DAU); 1252 δ (C-O-H, DAU); 1207 ν (C-O, glycosidic bond); 1167 ν (C-O, ester); 1068 ν_as_ (Si-O-Si, Si-O-C); 1047 ν (C-O, DAU); 1022 ν (Si-O-Si); 985 ν (C-H, alkyl); 984 ν (C-O-C, DAU); 839 δ (Si-C, C-O); 823 δ (in-plane ring PSF); 576 δ (Si-O); 556 ν (O-Si-O). λ_absorb.max_ = 502 nm in methanol.

To obtain iBuPOSSPSF photosensitizer, (3-mercaptopropyl)heptaisobutyl-POSS (iBuPOSS-SH) was first conjugated with allyl succinic anhydride via the thiolene addition reaction and then coupled to the PSF via an amide bond [32,39]. Briefly: allyl succinic anhydride (87 mg, 0.62 mmol) and (iBuPOSS-SH) (500 mg, 0.56 mmol) in the presence of AIBN in anhydrous toluene at 60 °C gave 519 mg of iBuPOSSAN (yield 90%). Anhydride substituted-POSS (310 mg, 0.30 mmol) was then added to phenosafranin hydrochloride (106 mg, 0.33 mmol) dissolved in 10 mL of anhydrous DMF in the presence of triethylamine (66 mg, 0.65 mmol). The reaction was carried out at room temperature for 48 h, then the solvent and unreacted amine ware evaporated. The product, iBuPOSSPSF (359 mg, 89% yield) was purified by multiple precipitation in hexane, dialyzed against methanol and then dried under vacuum. The NMR spectroscopic data are summarized in Table 1. Furthermoer, λ_absorb.max_ = 524 nm in methanol, *m*/*z* (TOF MS ESI) found: 1317.4620, molecular formula C_56_H_93_N_4_O_15_Si_8_S requires 1317.4512.

### 3.4. Antibacterial Photodynamic Studies In Vitro

The inoculum of bacteria was incubated in BHI medium at 37 °C for 18 h. After incubation, appropriate suspensions of micro-organisms were prepared in PBS buffer (pH = 7.4) with an optical density corresponding to 10^6^ CFU/mL. The suspensions of micro-organisms were introduced to 96-well microtiter plates containing the appropriate compounds in a final concentrations equivalent to 0.38 and 6.3 μM. Next, samples were incubated for 15, 30, 60 and 120 min at 37 °C and then irradiated with green light for 5 and 10 min. The custom constructed LED-based light source was used: emitting λ_max_ = 522 nm light with a radiosity of 10.6 mW/cm^2^ (at 65 mm distance) (FWDH (full width half maximum) 34 nm) (Cezos, Gdynia, Poland). After the illumination was completed, serial dilutions were made in PBS buffer (pH = 7.4) and plated on BHI agar. Incubation for 48 h at the appropriate temperature was performed and the micro-organisms grown on the plates were counted. Experiments in the dark were carried out simultaneously using the same time and temperature parameters.

### 3.5. Determination of Singlet Oxygen [^1^O_2_] Production

The protocol used to quantify the singlet oxygen yield was adapted from Fila et al. [60,61]. An experiment was performed for POSSPSF, iBuPOSSPSF and POSSPSFDAU in PBS buffer (pH = 7.4) with a singlet oxygen sensor green probe (SOSG) (500 µM), purchased from Thermo Fisher Scientific (Waltham, MA, USA). In the presence of a singlet oxygen, a sensor emits a green fluorescence similar to that of fluorescein (excitation/emission maxima ~504/525 nm). The sensor was prepared according to the procedure describe by the manufacturer. A measure of 90 µL of PBS buffer was introduced to the black 96-well plate and 5 µL of conjugates were added to estimate the final concentrations of 6.2, 1.6 and 0.4 µM. Next 5 µL of SOSG was added to each well (final concentration 5 µM), and the probes were incubated for 10 min in the dark and exposed to the green light (λ_em. max_ = 522 nm, 10.6 mW/cm^2^) for 5 minutes. Next, fluorescence was measured using an EnVision plate reader at excitation/emission wavelengths of 488/525 nm. The experiment was performed in three independent replicates.

### 3.6. Confocal Imaging of Bacterial

The cultures of *S. aureus* strain Newman, *S. aureus* MRSA 12673 and *E. coli* 12519 were grown in BHI broth until mid-log phase and then centrifuged. Next, the bacterial pellets were washed three times using PBS buffer (pH 7.4) and resuspended in the same buffer to achieve the optical density corresponding to 10^6^ CFU/mL. Bacterial cells were incubated with POSSPSF, iBuPOSSPSF and POSSPSFDAU at concentration equivalent to 0.38 μM for 1 h at 37 °C. After incubation, the cells were centrifuged, washed three times with PBS buffer and fixed on a glass slide. The localization of POSSPSF, iBuPOSSPSF and POSSPSFDAU in bacterial cells was observed by confocal microscopy imaging from Leica Microsystems (Wetzlar, Germany).

### 3.7. Evaluation of the Binding of POSSPSF, iBuPOSSPSF and POSSPSFDAU to Bacterial Cells

The mid-log phase *S. aureus* MRSA 12673 and *E. coli* 12519 cells were centrifuged, and the pellets were washed 3 times with PBS buffer (pH 7.4). Then, the pellets were resuspended in the same buffer reaching the optical density corresponding to 10^6^ CFU/mL. Bacterial cells were incubated with POSSPSF, iBuPOSSPSF and POSSPSFDAU at concentrations of 0.4, 1.6 and 6.2 µM for 1 h at 37 °C. They were then centrifuged, and the pellets and supernatants were separated. Then, the pellets were resuspended in the 200 µL of the PBS buffer (pH 7.4) and the intensity of fluorescence of both solutions (200 µL pellets and 200 µL supernatants) was measured by spectrophotometry at λ_max_ = 522 nm with a plate reader (Infinite M200 PRO, Tecan, Switzerland). The experiment was also carried out by changing the proportions for the fluorescence measurements. After centrifugation from 2 mL of bacterial suspension with photosensitizer, the bacteria were suspended in 2 mL of PBS buffer (pH 7.4), and 200 μL of bacterial suspension was collected and centrifuged. Then, 200 μL of supernatant and 200 μL of resuspended pellet was used for measurements. The results are presented as graphs with relative fluorescence intensity calculated to the fluorescence of photosensitizers solutions.

### 3.8. Transmission Electron Microscopy Assay

TEM was performed as described previously [62]. Tested samples were adsorbed on formvar–carbon-coated 200-mesh copper grids. Negative staining was performed with 2% uranyl acetate. The grids were analyzed using a transmission electron microscope, Tecnai G2 Spirit BioTWIN (FEI Inc., Hillsboro, OR, USA), at 120 kV, at the Laboratory of Electron Microscopy, University of Gdansk.

### 3.9. Statistical Analysis

All experiments were performed in triplicates, in three independent experimental sets. The graphics and the data were constructed and analyzed statistically by means ± SD.

## 4. Conclusions

In summary, we constructed light-triggered ROS-responsive phenosafranin-polyhedral oligomeric silsesquioxane (POSS) conjugates as water-soluble nanoparticle for photodynamic antibacterial therapy of gram-positive and gram-negative bacteria. The positively charged conjugates composed of covalently linked inorganic POSS cage and phenosafranin dye may increase the accumulation of the photosensitizer inside bacterial cells, as observed with POSSPSF conjugate, or the accumulation in the bacterial cell membrane or cell wall, as observed with iBuPOSSPSF and POSSPSFDAU. Bacterial cells were eradicated by ROS produced upon irradiation of the covalent conjugates with light (λ_em. max_ = 522 nm, irradiation = 10.6 mW/cm^2^) that can kill the bacteria by destruction of cellular membranes, intracellular proteins and DNA through the oxidative damage of bacteria. The completed eradication of bacteria was obtained after light irradiation for 5 min with *S. aureus* and *E. coli* bacteria cells, incubated with very low concentration of POSS-photosensitizers (0.38 µM). In contrast, no dark toxicity of the conjugates was observed.

POSS decorated with the desired phenazine, and anthracycline moieties was performed according to a synthetic procedure based on two click-type reactions: thiolene addition and amide bond synthesis by reaction of NH_2_-substituted PSF with highly reactive succinic anhydride-POSS. Both reactions ensure short synthesis times, selectivity and high yields of pure organic-inorganic nanoparticles. The results showed that these new POSS conjugates could improve the efficiency of second-generation antimicrobial photosensitizers, a very important therapeutic advance in the face of today’s bacterial and viral threats.

## Figures and Tables

**Table 1:** ^1^H, ^13^C NMR shift assignment of PSF-photosensitizers in DMSO-d_6_.

	iBuPOSSPSF		POSSAN ^a)^		POSS-PSF		POSS-PSF-DAU				
No.	^1^H, δ (ppm)	^13^C, δ (ppm)	^1^H, δ (ppm)	^13^C, δ (ppm)	^1^H, δ (ppm)	^13^C, δ (ppm)	^1^H, δ (ppm)	^13^C, δ (ppm)	No.	^1^H, δ (ppm)	^13^C, δ (ppm)
a	0.68 *(2H)*	10.5	0.13 *(6H)*	0.6	0.08 *(6H)*	0.17	0.07 *(6H)*	0.19	11	7.64 *(1H)*	120.9
b	2.46 *(2H)*	33.1	0.63 *(2H)*	16.9	0.53 *(2H)*	17.9	0.51 *(2H)*	17.80	12	7.82 *(1H)*	134.0
c	1.69 *(2H*)	25.5	1.46 *(2H)*	20.18	1.28 *(2H)*	19.9	1.33 *(2H)*	20.61	13	7.91 *(1H)*	120.7
d1 d2	1.98 *(2H)*	35.2	1.68 *(1H)* 1.91 *(1H)*	34.0	1.40–1.54 *(2H)*	35.0	1.36 *(1H)* 1.67 *(1H)*	35.13	14 14a	-	160.7 119.0
e	2.98 *(1H)*	42.2	3.11 *(1H)*	40.27	3.18 *(1H)*	41.5	2.98 *(1H)*	40.09	15, 15’ 15a	-	186.5 110.7
f1 f2	2.72 *(2H)*	35.7	2.64 *(1H)* 3.06 *(1H)*	34.21	2.31–2.40 *(2H)*	36.6	2.61 *(1H)* 2.84 *(1H)*	35.20	16, 16’	13.34 *(1H)* 14.09 *(1H)*	154.5
g, h	-	173.0	-	173.8, 170.3	-	173.8	-	173.7	17	4.94 *(1H)*	72.1
1, 1’	8.11 *(1H*, *J = 9 Hz)* 7.93 *(1H*, *J = 9 Hz)*	133.8			8.09 *(1H*, *J = 9 Hz)* 7.92 *(1H, J* = 9 Hz)	136.6 133.8	8.11 *(1H)* 7.91 *(1H)*	136.9 127.7	18	2.02 *(2H)* 1.80	35.2
2, 2’	7.62 *(1H)* 7.20 *(1H*, *J = 9 Hz, J = 2 Hz)*	125.9 121.0			7.63 *(1H)* 7.21 *(1H,J = 9, J = 2 Hz)*	125.1 120.1	7.57 *(1H)* 7.22 *(1H)*	120.9	19	5.58 *(1H)*	75.3
3, 3’	-	157.2 141.5			-	157.0 141.6	-	157.14	20	2.24 *(2H)* 2.46	31.5
4, 4’	5.99 *(1H)* 5.85 *(1H*, *J = 2 Hz)*	93.4 92.7			5.91 *(1H)* 5.81 *(1H, J = 2 Hz)*	93.5 92.7	6.01 *(1H)* 5.94 *(1H)*	93.5 92.8	21	-	211.8
5, 5’	-	134.2			-	134.2	-	134.5	22	2.21 *(3H)*	24.1
6	-	136.1			-	136.1	-	136.0	23	3.99 *(3H)*	56.6
7, 7’	7.63 *(H + H)*	127			7.65 *(H + H)*	127.5	7.69 *(H + H)*	130.0	24	5.25 *(1H)*	100.2
8, 8’	7.81 *(H + H)*	131.0			7.7–7.9 *(H + H)*	131.5	7.78 *(H + H)*	133.7	25	1.51 *(2H)* 1.86	29.6
9	7.8 *(1H)*	130.6			7.8 *(1H)*	130.2	7.83 *(1H)*	136.0	26	3.87 *(1H)*	45.1
10, 10’	-	134.8	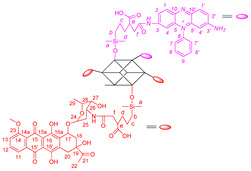 POSSPSFDAU			134.8			27	3.56 *(1H)*	66.8
a1	1.56 *(2H)*	22.5				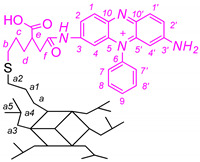 iBuPOSSPSF			28	4.27 *(1H)*	70.0
a2	2.44 *(2H)*	30.1							29	1.19 *(3H)*	17.1
a3	0.57 *(2H)*	21.8							NH	7.50	-
a4	1.78 *(1H)*	23.3									
a5	0.91 *(6H)*	25.2									

a) Recorded in CDCl_3_.

## Data Availability

The data presented in this study are available on request from the corresponding authors.

## Supplementary Materials

The following are available online at https://www.mdpi.com/article/10.3390/ijms222413373/s1.
